# Understanding the competing pathways leading to hydropyrene and isoelisabethatriene

**DOI:** 10.3762/bjoc.18.97

**Published:** 2022-08-04

**Authors:** Shani Zev, Marion Ringel, Ronja Driller, Bernhard Loll, Thomas Brück, Dan T Major

**Affiliations:** 1 Department of Chemistry and Institute for Nanotechnology & Advanced Materials, Bar-Ilan University, Ramat-Gan 52900, Israel; 2 Werner Siemens Chair of Synthetic Biotechnology, Department of Chemistry, Technical University of Munich, Lichtenbergstraße 4, 85748 Garching, Germany; 3 Institute for Chemistry and Biochemistry, Structural Biochemistry Laboratory, Freie Universität Berlin, Takustr. 6, 14195 Berlin, Germany; 4 Department of Molecular Biology and Genetics, Aarhus University, Danish Research Institute of Translational Neuroscience – DANDRITE, Universitetsbyen 81, 8000 Aarhus C, Denmark

**Keywords:** diterpenes, enzyme mechanism, quantum mechanics, terpene synthases, thermodynamic and kinetic control

## Abstract

Terpene synthases are responsible for the biosynthesis of terpenes, the largest family of natural products. Hydropyrene synthase generates hydropyrene and hydropyrenol as its main products along with two byproducts, isoelisabethatrienes A and B. Fascinatingly, a single active site mutation (M75L) diverts the product distribution towards isoelisabethatrienes A and B. In the current work, we study the competing pathways leading to these products using quantum chemical calculations in the gas phase. We show that there is a great thermodynamic preference for hydropyrene and hydropyrenol formation, and hence most likely in the synthesis of the isoelisabethatriene products kinetic control is at play.

## Introduction

Terpenes constitute a ubiquitous class of natural molecules that are synthesized by terpene synthases (TPS). TPS generate a plethora of terpenes employing rich carbocation chemistry from a very limited number of substrates, known as geranyl pyrophosphate (GPP), farnesyl pyrophosphate (FPP), and geranylgeranyl pyrophosphate (GGPP), to produce mono-, sesqui-, and diterpenes, respectively. The formation of terpenes relies on an assortment of carbocation steps like cyclization, methyl migrations, rearrangements, proton or hydride transfers, hydroxylations, and epoxidations. TPS and downstream functionalizing enzymes, like P450s, together produce more than 80,000 known terpenes and terpenoids [1–3].

Hydropyrene synthase (HpS) from *Streptomyces clavuligerus* generates a mixture of diterpenes named hydropyrene (HP) (52%) and diterpenoid named hydropyrenol (HPol) (26%) as its main GGPP cyclization products, along with two minor compounds, namely the isoelisabethatrienes (IEs) A (13%) and B (9%), respectively. Interestingly, the elisabethatriene diterpene macrocycle and its isoforms can act as biosynthetic precursors of the bioactive compounds erogorgiaene and pseudopterosin, having antibiotic and anti-inflammatory activities, respectively [4–5]. Unexpectedly, a single active site mutation, M75L, significantly shifts the product distribution and IE A becomes the dominant product (44%) in this enzyme variant [6].

**Figure 1:**
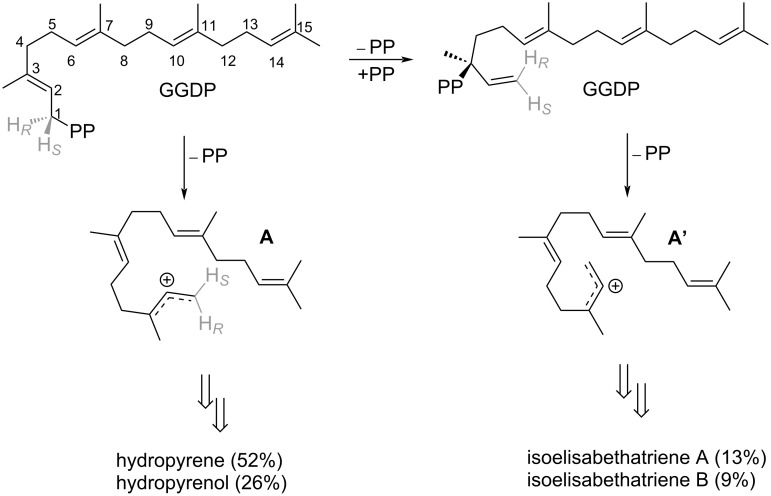
Summary of yields of HP and IE products in hydropyrene synthase.

As suggested by Rinkel et al., both routes (HP and IE routes) proceed from the same substrate (GGPP) with an initial C1–C10 cyclization. In other TPS enzymes, the initial fold of GGPP in the active site can result in different initial cyclization, for example C1–C6, C1–C7, C1–C10, C1–C11, C1–C14, and C1–C15. The main difference between the two pathways to HP and IE, is that in the HP pathway C1–C10 cyclization occurs immediately, while in the IE pathway a substrate transoid to cisoid conformational change occurs, shifting the covalent attachment point (i.e., C1 to C3) between the substrate hydrocarbon and the pyrophosphate group [7] (Figure 1). Presumably, this isomerization is responsible for a slightly different substrate fold inside the active site, hence shifting the product distribution in favor of the IE products in certain enzyme variants rather than the HP products.

Oxidation of IEs A and B by lipases results in the formation of the advanced pseudopterosin (P) precursor erogorgiaene and (1*R*)-epoxyelisabetha-5,14-diene (EED), respectively [6–7]. Ps, marine amphilectane-type diterpenoids from the gorgonian coral *Antillogorgia elisabethae*, feature superior anti-inflammatory properties which render them innovative target compounds for drug development [8–9]. Hence, increasing the IE products at the expense of the HP products is an important biotechnology mission for sustainable supply of the latter. In order to modulate the IE and HP enzyme pathways accordingly, it is important to understand the factors determining both synthetic routes.

In the current work, we focus on the mechanistic details of the HP and IE pathways using computational methods in the gas phase. Gas-phase studies have been crucial in understanding terpene chemistry [10–22]. This work sheds light on the thermodynamic and kinetic parameters of the inherent chemistry in these reactions and also points to some understanding of the possible thermodynamic and kinetic control in the enzyme.

## Results

### Reaction mechanism

**Scheme 1:**
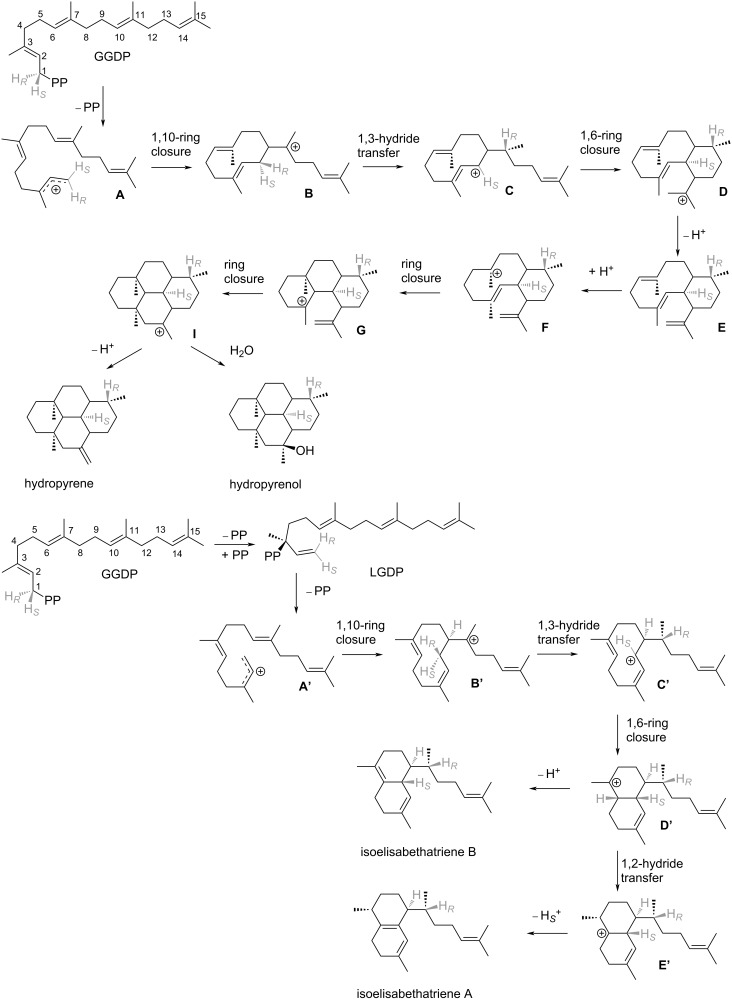
Proposed mechanism for HP and IE routes.

**Figure 2:**
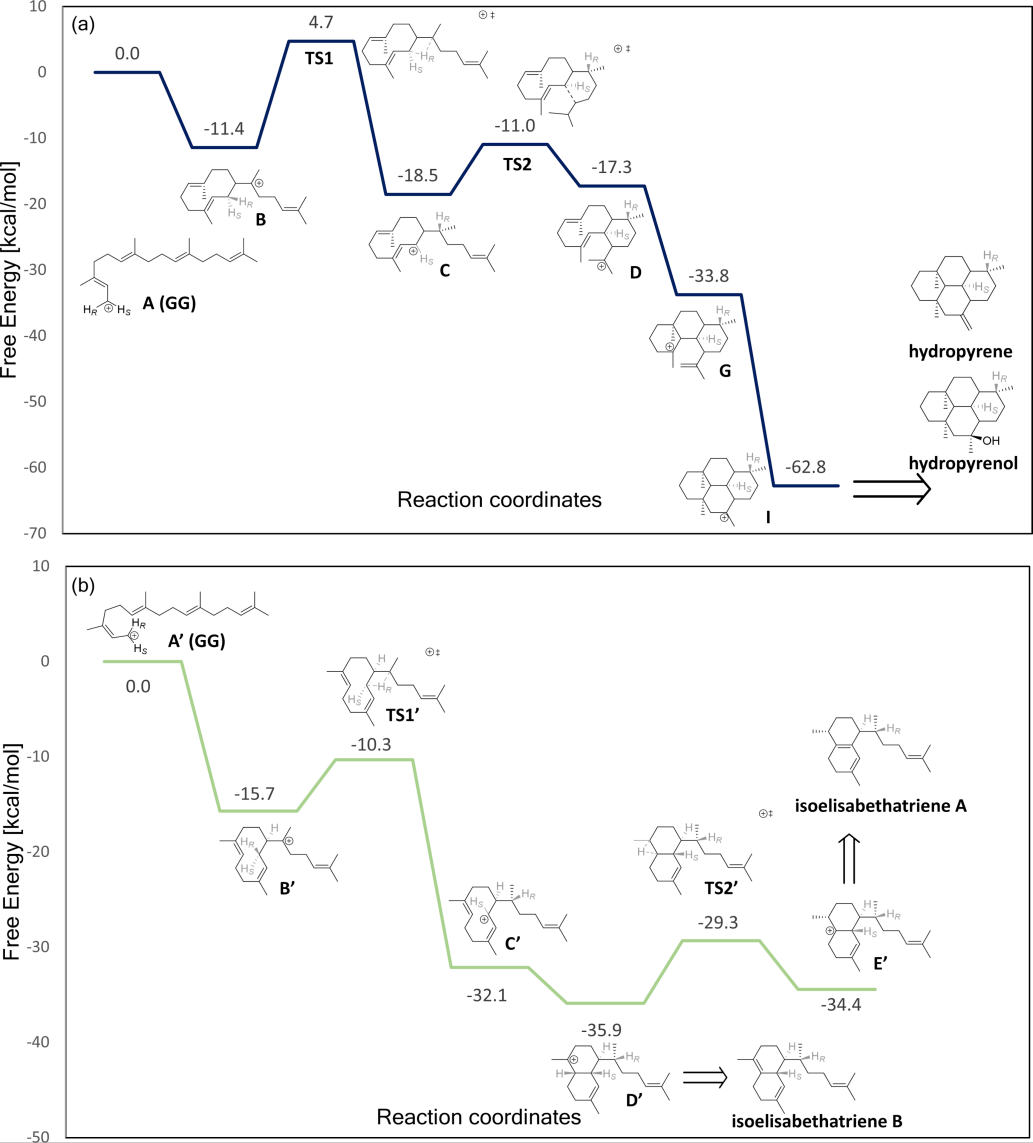
Free energy profile of hydropyrene cation (a), and IE cation (b) formation in the gas phase. The free energy of cation **A** and **A’** is set to zero. Bonds breaking/forming in the transition states are marked by dotted lines. All calculations were performed in the gas phase at the M06-2X/6-31G+(d,p) level of theory.

To better understand the HP and IE reaction pathways, we performed quantum mechanics (QM) calculations using density functional theory (DFT). We studied the inherent chemistry of the reaction leading to HP and IE using gas-phase calculations. This provided the free energy of distinct carbocation intermediates and transition states along the proposed reaction path leading to products in the gas phase. The gas phase is a natural choice as a reference environment for terpene synthases [10–12,15–16,21–25]. The proposed reaction mechanisms yielding HP and IE and are presented in Scheme 1, while the reaction free energy profile is presented in Figure 2. Here, we modeled the transformations **A→I** (HP) and **A’→E’** (IE). The gas-phase calculations commenced with geranylgeranyl cation (**A** and **A’**) in a fully extended form.

### HP pathway

The HP gas-phase pathway commences with a C1–C10 cyclization, which yields cation **B**, which is more stable than **A** by −11.4 kcal/mol. A subsequent 1,3-hydride transfer results in an allyl cation (**C**), which is −18.5 kcal/mol more stable than **A**. The barrier for the 1,3-hydride transfer is 16.2 kcal/mol for **B→C**. Subsequently, the double bond on C14–C15 reacts with the cationic charge on C1 to form intermediate **D**, which is slightly less stable than **C** (−17.3 kcal/mol) In the enzyme environment intermediate **D** deprotonates to form intermediate **E**, while intermediate **E** is re-protonated to form intermediate **F**, which immediately transforms to intermediate **G (**i.e., **F** is not stable**)**. **G** is significantly more stable than **D** (by −16.5 kcal/mol). **G** then transforms to the very stable 4-ring intermediate **I** without any free energy barrier. The deprotonation and re-protonation steps are not included in our calculations. The overall exergonicity of this process which transforms four π-bonds to σ-bonds, with accompanying gains in intramolecular dispersion interactions, is −62.8 kcal/mol.

### IE pathway

As described above both pathways commence with a C1–C10 cyclization. However, in the IE pathway a preliminary isomerization step occurs via rotation around the C2–C3 bond, transforming from the *trans* to the *cis* form. In the enzyme this process occurs with the help of a pyrophosphate group. The C1–C10 cyclization yields cation **B’**, which is more stable than **A’** by −15.7 kcal/mol. A subsequent 1,3-hydride transfer results in an allyl cation (**C’**, −32.1 kcal/mol relative to **A’**), with a barrier of 5.4 kcal/mol. Cation **C’** collapses into **D’** via a barrierless 1,6-ring closure (Δ*G*_r_ of −35.9 kcal/mol relative to **A’**). **D’** can be deprotonated to yield IE B or conversely may undergo a 1,2-hydride transfer, forming carbocation **E’** (Δ*G*_r_ of −34.4 kcal/mol relative to **A’**). This transformation has a Δ*G*^‡^ of 6.6 kcal/mol. Cation **E’** may then be deprotonated to form IE A. Overall, the exergonicity for the formation of IEs A/B from carbocation **A’** is significant, due to the exchange of two π-bonds for σ-bonds, as well as gain in dispersion interactions on folding of the extended geranylgeranyl cation.

## Discussion

**Figure 3:**
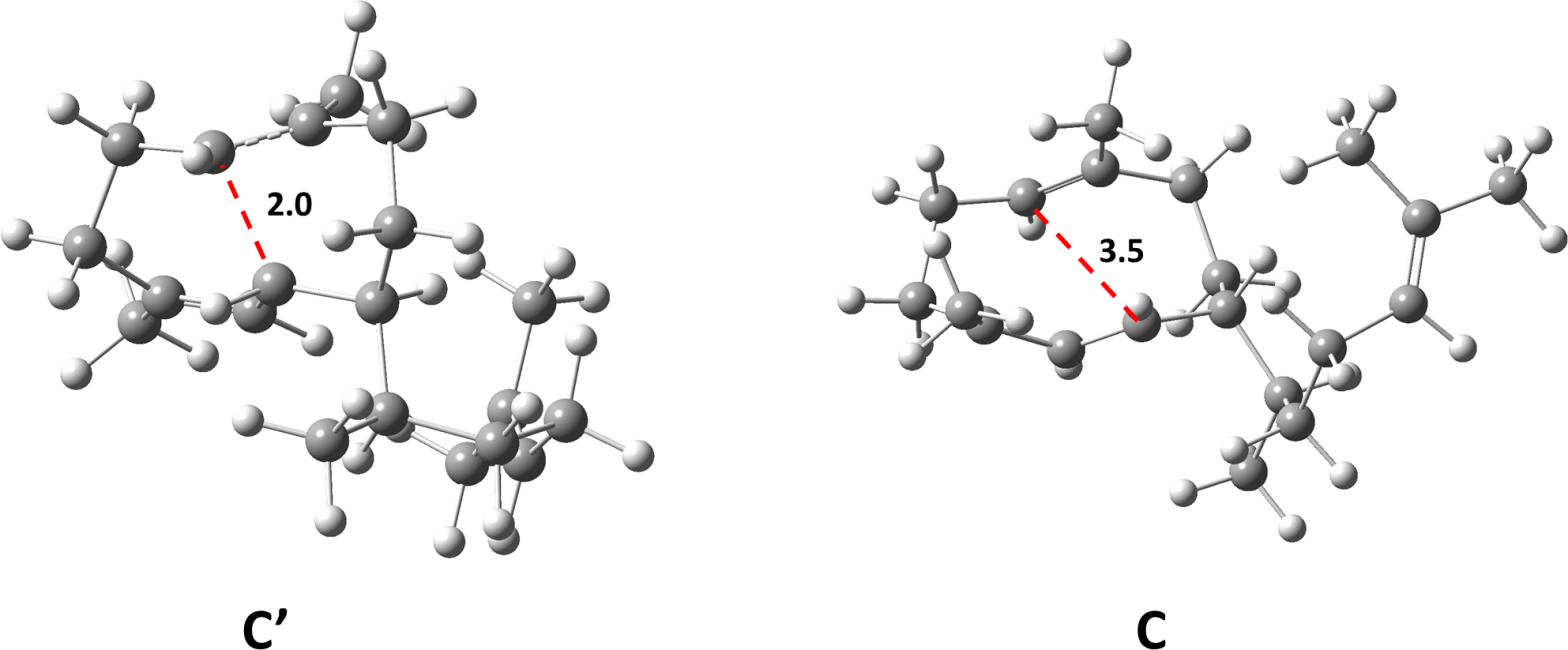
Structures of intermediates **C**‘ and **C**. The distance between the double bond and the cation in intermediate **C‘** is 2.0 Å while in **C** the distance is 3.5 Å.

Although the current calculations were performed in the gas phase without inclusion of the enzyme environment, we may still generate some hypotheses regarding the enzymatic process. First, considering the similar free energy of IEs A and B and the small kinetic barrier separating them, these isomers may exist in equilibrium in the enzyme active site. The relative amount of IEs A and B may then be determined by their proximity to an active site base, as has been observed in other cases, like taxadiene synthase [26]. Second, the chemical changes occurring up to intermediates **C** and **C’** in both routes are similar and diverge in the transformation of **C→D** and **C’→D’**. Here, we suggest that the ring closure in the IE pathway (**C’→D’**) occurs immediately due to the *cis* conformation and the short distance between the cation and the double bond (≈2 Å), which could be due to the active site substrate fold. In intermediate **C**, the *trans* conformation and possibly slightly different substrate fold, results in an alternative reaction route being followed (Figure 3). Lastly, considering the huge thermodynamic preference for the HP pathway, it is unlikely that a thermodynamic equilibrium exists between this pathway and the IE pathway. Rather, an equilibrium may exist between GGDP and LGDP, but once cyclization commences, the reactions will proceed until completion along their respective pathways. Hence, the difference in the product profile in WT and enzyme variants may be largely due to different folding of the initial substrate. Future in-enzyme studies can shed light on the preferred folding of GGDP inside the WT and variant enzymes and potentially the roles specific active site residues play during catalysis [23–24,27].

## Conclusion

In the current work we performed gas-phase quantum chemistry calculations for the competing reaction pathways leading to IEs A and B as well as hydropyrene and hydropyrenol. In the former two reactions there is an exchange of two π-bonds for σ-bonds, resulting in a total exergonicity of −34.4 and −35.9 kcal/mol, respectively. In the latter two reactions which replace four π-bonds for σ-bonds and share a common final carbocation, the exergonicity is −62.8 kcal/mol. These values reflect the energetics of exchanging π-bonds for σ-bonds. In spite of the current calculations being performed in the gas phase, we may still generate some propositions regarding the enzymatic process. First, considering the similar free energy of IEs A and B and the low barrier between them, IEs A and B may exist in equilibrium in the enzyme active site. The proximity to an active site base may then determine the relative amount of IEs A and B. Second, it is unlikely that a thermodynamic equilibrium exists between the HP and IE pathways, due to the significant free energy barriers required for reverse barriers in the enzyme. Rather, an equilibrium may exist between GGDP and LGDP.

## Experimental

### Dynamics and Monte Carlo simulations

We generated conformers using simulated annealing (SA) molecular dynamics followed by SA Monte Carlo simulation using CHARMM [28]. Force field parameters were generated using CGenFF [29] and an in-house code which modifies parameters for cations from existing parameters for neutral molecules. For each intermediate in the reaction mechanism (path HP and IE) we created 100 conformers which were subsequently clustered (a cluster width of 1.0 Å was used). For each unique conformer we performed QM calculations and then chose the lowest energy conformer as representative of each carbocation intermediate in the mechanism. Hence, each intermediate state is represented by a single conformer, which is the lowest energy conformer found.

### Quantum chemistry calculations

Optimizations and subsequent frequency calculations were performed using the Gaussian 16 program [30] at the M06-2X/6-31G+(d,p) level of theory [31–32]. This combination has been employed previously to TPS reactions [17–18,20,24–25,33–36]. The Gibbs free energies were calculated within the standard harmonic approximation and at a pressure of 1 bar and temperature of 298 K. We note that for some reaction steps, a transition state could not be located.

## Supporting Information

File 1 All coordinate files for intermediates and TS structures.
